# Exploring safety of down‐titrating diuretics in heart failure management

**DOI:** 10.1002/ejhf.3714

**Published:** 2025-07-02

**Authors:** Chrit B.G. Geraeds, Anke Bruninx, Andre L.A.J. Dekker, Arantxa Barandiarán Aizpurua, Inigo Bermejo, Hans‐Peter Brunner‐La Rocca

**Affiliations:** ^1^ Department of Cardiology CARIM, Maastricht University Medical Centre Maastricht The Netherlands; ^2^ Department of Radiation Oncology (MAASTRO) GROW Research Institute for Oncology and Reproduction, Maastricht University Medical Centre Maastricht The Netherlands; ^3^ Cardiology, Maastricht University Medical Centre Maastricht The Netherlands

**Keywords:** Heart failure, Diuretics, Therapy, Safety

## Abstract

**Aims:**

Loop diuretics are widely used in heart failure (HF) for symptom relief. Guidelines advise treating patients with the lowest possible dose of loop diuretics to maintain euvolaemia, based on expert opinion only. However, data on the safety of this practice are scarce. This analysis aims to investigate the clinical course after changing loop diuretics in stable HF patients.

**Methods and results:**

A post‐hoc analysis from the TIME‐CHF study was conducted in 622 patients. Daily loop diuretic doses were meticulously recorded, resulting in 11 035 dose evaluations assessed. The frequency of relevant events within 30 days (hospitalization, death, dose change) following an up‐titration, down‐titration, or no change in diuretic therapy was calculated. Risk of hospitalization and deaths 30 days after down‐titration was estimated after adjusting for congestion level. At baseline the cohort consisted mostly of males (59%), with a mean age of 77 years. Patients were highly symptomatic with 76% classified as New York Heart Association class III or IV. Within 30 days after a down‐titration, diuretic dose necessitated an increase in 30.4% of cases, compared with 20.7% following up‐titration and 8.0% on a stable dose. Hospitalization and death were significantly more frequent following down‐titration (3.4% and 2% within 30 days, respectively) than with a stable dose (1% and 0.6%, *p* < 0.001). Similar hospitalization rates were observed after up‐titration. The risk of hospitalization doubled and of death tripled after down‐titration in patients with similar congestion levels.

**Conclusions:**

There is a significant risk that reduction in diuretic dose requires restart or increase of diuretics within a short period. Additionally, there may be a significant risk associated with diuretic dose reduction. Therefore, HF patients need to be closely monitored after down‐titration of loop diuretic therapy.

## Introduction

Heart failure (HF) is a chronic disease that is characterized by an array of debilitating signs and symptoms.^1^ The disease manifests, among others, through episodes of sudden worsening of symptoms due to fluid retention, called decompensation.^2^ Treatment of these episodes often requires close‐monitor care and the use of (intravenous) diuretic therapy. It is the most prominent cause of hospital admission in elderly patients.^3^, ^4^, ^5^ This escalating trend in hospitalizations coupled with the growing prevalence of HF has led the disease to become one of the leading causes in healthcare costs worldwide.^6^, ^7^, ^8^

In the management of HF, the core objectives are to stabilize the disease, relieve patients of their symptoms, and prevent or minimize episodes of decompensation.^9^ Achieving these goals involves the use of disease‐modifying therapy, that is, the ‘fantastic four’ in patients with reduced left ventricular ejection fraction (LVEF), in addition to sodium–glucose cotransporter 2 (SGLT2) inhibitors in HF with preserved LVEF, which have demonstrated efficacy across the entire range of LVEF. Moreover, loop diuretics play a pivotal role as the main treatment for congestion.^1^ The most recent HF guidelines advise treating patients with the lowest possible dose of loop diuretics to reach and maintain euvolaemia.^1^ In other words, down‐titration of the dose of loop diuretics should be considered after euvolaemia is achieved. However, this recommendation is based on expert opinion only and lacks scientific evidence. Conversely, the implementation of invasive monitoring through implantable devices in patients with chronic HF revealed a predominance of dose increases over decreases, resulting in reduced hospitalization rates.^10^, ^11^ Therefore, it remains unclear how to optimize diuretic therapy adjustments and whether a reduction in diuretic therapy can be considered safe.

To gain further insight into the effects of changes in diuretic therapy, we conducted a retrospective post‐hoc analysis using data from the Trial of Intensified versus standard Medical therapy in Elderly patients with Congestive Heart Failure (TIME‐CHF), which provides daily information on all drugs used including doses during 18 months. Our primary aim was to provide a more data‐driven perspective on the effects and implications of changes in loop diuretic therapy in HF patients.

## Methods

### Data and subjects

The TIME‐CHF database was established between 2003 and 2008 in Switzerland and Germany. The design and main results of the study have previously been published.^12^, ^13^, ^14^ In short, the comparison of N‐terminal pro‐B‐type natriuretic peptide (NT‐proBNP)‐guided therapy versus symptom‐based management of HF patients was investigated in both patients with reduced and preserved LVEF. The study consisted of 622 HF patients, aged over 60 years, exhibiting symptoms according to the New York Heart Association (NYHA) of class II or higher and elevated NT‐proBNP levels. Clinical evaluations took place at baseline and after 1, 3, 6, 12, and 18 months. Throughout the study period, a large amount of data including detailed drug information was collected, which included the daily dose taken by patients of all drugs including loop diuretics, changes of drugs and the reasons for adjusting the therapy. The loop diuretic dose was expressed as furosemide equivalent, where 10 mg of torasemide or 1 mg of bumetanide was considered equivalent to 40 mg of furosemide. Importantly, dose changes were recorded not only at, but also between the study visits. With this information, we were able to create a detailed dataset showing the daily loop diuretic dose per patient for the duration of their study participation of 18 months.^12^ A previous retrospective analysis of TIME‐CHF data has established a congestion score for TIME‐CHF patients that is informative of the severity of a patient's HF. This so‐called ‘clinical congestion index’ is composed of the following signs and symptoms: hepatomegaly, NYHA class ≥III, oedema, jugular vein distention, orthopnoea, rales, and paroxysmal nocturnal dyspnoea, and scores a patient one point per sign/symptom.^15^

The TIME‐CHF study was approved by the Ethics Committee of Basel, Switzerland, and of all local centres and by the Swiss and German regulatory bodies. No additional ethical approval was acquired for this project.

### Pre‐processing

All patients retrospectively suspected of palliative care were excluded from the analysis, since this might lead to an overestimation of mortality following down‐titration. These patients were characterized as follows: the last dose change was a down‐titration to 0 mg (in other words, cancellation of the loop diuretic therapy) and death occurred within 7 days after cancellation of the loop diuretic therapy.

### Statistical analyses

Using this dataset, we undertook two different analyses. Firstly, we segmented each patient's follow‐up time into intervals lasting until the next event (dose change, hospitalization, or death) or 30 days of dose maintenance, whichever occurred first. We then calculated 30 day frequencies of hospitalization for worsening of HF, death or dose changes subsequent to down‐titration, up‐titration, dose maintenance or hospital discharge. To quantify the uncertainty around these estimates, we calculated bootstrapped 95% confidence intervals (CI). We excluded from this analysis the time patients spent in the hospital due to worsening HF. Consequently, intravenous diuretics administered during hospitalization were not considered, and only diuretic dose adjustments made in the outpatient setting were included. Secondly, we aimed to assess whether the difference in outcomes in patients whose dose was down‐titrated remained after adjusting for confounding factors (i.e. patients whose dose was down‐titrated being more severely ill than those who did not). In this analysis, we looked at the 30 days after each evaluation, excluding the final assessment at 18 months, after which dose information was not collected. We fitted models to predict the probability of congestive HF‐related hospitalization and mortality utilizing the congestion score and whether the patient's dose had been down‐titrated after the visit or had remained unchanged as predictive factors. Patients whose dose was up‐titrated were excluded from this analysis to prevent the effect of up‐titrations in the comparator group obfuscating the assessment of the effect of down‐titration. Our objective in adjusting for the congestion score was to minimize potential confounding effects. We then used the parametric g‐formula to estimate the causal effect of down‐titration versus no dose change on congestive HF‐related hospitalization and mortality.^16^ This method involved developing predictive models to assess the probability of hospitalization and mortality, incorporating confounders such as the congestion score, and the exposure variable, that is, down‐titration of loop diuretics. Counterfactual outcomes were simulated for each individual in the study population under different exposure scenarios, allowing for the estimation of causal effects. By adjusting for potential confounding factors and comparing simulated outcomes, the parametric g‐formula estimates the impact of down‐titration of loop diuretics on the outcomes of interest. We performed the statistical analysis using IBM SPSS statistics version 28.0.0.0 and R 4.2.1. statistical software.^17^

## Results

At baseline, our study population consisted of slightly more men, accounting for 59% (*n* = 369) of the population with a mean age of 77 ± 8 years. Most patients were highly symptomatic with 76% (*n* = 473) of patients having a NYHA class ≥III and NT‐proBNP levels were significantly elevated. Patients suffered from a broad range of comorbidities. Full demographics and baseline characteristics are shown in *Table* *1*.

**Table 1:** Demographics, baseline characteristics, and comorbidities

Demographics	
Patients, *n*	622
Age, years, mean ± SD	77 ± 8
Male sex, *n* (%)	369 (59)
BMI, kg/m^2^, mean ± SD	26 ± 4
Preserved LVEF >45%, *n* (%)	123 (20)
Baseline characteristics	
Systolic blood pressure, mmHg, median [IQR]	120 [110–135]
Diastolic blood pressure, mmHg, median [IQR]	70 [65–84]
Heart rate, bpm, median [IQR]	74 [65–84]
NYHA class, *n* (%)	
II	149 (24)
III	388 (62)
IV	85 (14)
Fatigue, *n* (%)	534 (86)
Angina, *n* (%)	125 (20)
Orthopnoea, *n* (%)	422 (68)
Nocturia, *n* (%)	530 (85)
Oedema, *n* (%)	260 (42)
Orthostasis, *n* (%)	292 (47)
Dizziness, *n* (%)	195 (31)
Syncope, *n* (%)	47 (8)
Rales, *n* (%)	280 (45)
Jugular vein distension, *n* (%)	372 (60)
NT‐proBNP, pg/ml, median [IQR]	3836 [1916–6905]
Creatinine, μmol/L, median [IQR]	108 [87–140]
LVEF, %, median [IQR]	23 [25–42]
Comorbidities, *n* (%)	
Coronary artery disease	400 (64)
Hypertension	462 (74)
Hypercholesterolaemia	303 (49)
Kidney disease	355 (57)
COPD	124 (20)
Diabetes mellitus	222 (36)
Stroke/TIA	98 (16)
Cancer	86 (14)

BMI, body mass index; COPD, chronic obstructive pulmonary disease; IQR, interquartile range; LVEF, left ventricular ejection fraction; NT‐proBNP, N‐terminal pro‐B‐type natriuretic peptide; NYHA, New York Heart Association; SD, standard deviation; TIA, transient ischaemic attack.

*Table* *2* shows the occurrence frequencies of various events subsequent to down‐titration, up‐titration, or maintenance of diuretic doses, as well as hospital discharge. Following down‐titration, up‐titration was deemed necessary within 30 days in 30.4% (95% CI 27.7–32.7%) of cases. In comparison, down‐titration was followed by another down‐titration in 16.3% (95% CI 14.2–18.4%) of cases. The 30 day mortality rate subsequent to down‐titration was 2.0% (95% CI 1.3–2.8%), compared to 1.2% (95% CI 0.7–1.8%) following up‐titration, and 0.6% (95% CI 0.5–0.8%) with stable diuretic doses (*p* < 0.001). Within 30 days after hospital discharge, 6.1% (95% CI 3.1–10.4%) of the patients deceased. The frequency of hospital admission after down‐titration was similar to that after up‐titration (3.4% vs 3.6%) and higher than after dose maintenance (1.0%, 95% CI 0.8–1.3%). The *Graphical Abstract* summarizes these findings. These results appear to be independent of ejection fraction as investigating both patients with LVEF <45% and LVEF >45% separately shows similar results. These results are displayed in online supplementary *Tables*.

**Table 2:** Frequency of events (in percentages) in 30 day intervals after dose changes, hospital discharge, or dose maintenance

After	*n*	Deceased	Hospital admission	Up‐titration	Down‐titration	No event
Dose maintenance	8177	0.6 (0.5–0.8)	1.0 (0.8–1.3)	8.0 (7.4–8.6)	7.6 (7.1–8.2)	82.7 (81.9–83.6)
Down‐titration	1327	2.0 (1.3–2.8)	3.4 (2.4–4.4)	30.4 (27.7–32.7)	16.3 (14.2–18.4)	48.0 (45.3–50.7)
Up‐titration	1368	1.2 (0.7–1.8)	3.6 (2.6–4.7)	20.7 (18.6–23)	34.1 (31.5–36.6)	40.5 (37.9–43.2)
Hospital discharge	163	6.1 (3.1–10.4)	6.7 (3.1–11)	19.6 (13.5–25.8)	17.2 (11.7–22.7)	50.3 (42.9–58.3)

Bootstrapped 95% confidence intervals in parenthesis.

The reason for change in diuretic therapy was coded in all cases. Up‐titration was related to signs and symptoms of decompensation in 95% of cases. In 2% of cases, up‐titration was solely related to the study protocol in the NT‐proBNP‐guided group,^12^ and in 1% no specific reason was mentioned. Down‐titration was done in 60% of cases if considered less was needed based on clinical course, signs and symptoms. In 11% of cases, worsening renal function was the reason for reducing diuretic therapy, followed by 13% due to hypotension, and in 6% diuretic therapy was reduced without mentioning a specific reason. Other incidental reasoning for adjusting diuretic therapy includes the presence of cough, infection, liver dysfunction, and other comorbidities.

For the second analysis, we fitted regression models to estimate the probability of HF‐related hospitalization and death within 30 days after a follow‐up visit using two predictors: the patient's congestion score at the evaluation and a binary variable determining whether a patient's dose had been down titrated or had remained the same. The number of samples to fit the model was 1786 with 34 hospitalizations for HF and 16 deaths. The models (summarized in *Table* *3*) show that even after adjusting for congestion score, down‐titration was associated with a higher probability of hospitalization (*p* = 0.03) and death (*p* = 0.06), depicted by a positive beta‐coefficient in the model showing this dose intervention is associated with an increased likelihood of these outcomes. Using the parametric g‐formula, the estimated risk of hospitalization was more than twice and death three times higher after down‐titration compared with no dose change as shown in *Table* *4*.

**Table 3:** Summary of models to estimate the risk of hospitalization for congestive heart failure and death

	Estimate	Standard error	*p*‐value
Hospitalization due to worsening of CHF			
Intercept	0.001	0.004	0.8516
Down‐titration	0.023	0.011	0.0342
Congestion score	0.010	0.002	<0.001
Death			
Intercept	−0.001	0.002	0.740
Down‐titration	0.013	0.007	0.0625
Congestion score	0.005	0.001	<0.001

CHF, congestive heart failure.

**Table 4:** Risk of hospitalization and death 30 days after down‐titration or dose change as estimated with the parametric g‐formula

	No dose change	Down‐ titration	Difference
Hospitalization due to worsening of CHF	0.015	0.038	0.023
Death	0.006	0.019	0.013

CHF, congestive heart failure.

## Discussion

Our findings show that approximately one‐third of patients require swift reinitiation or an increase of the dose after down‐titration and both hospitalization and mortality were increased. Thus, caution is advisable if down‐titration is intended. Current recommendations by guidelines are mostly based on expert opinion with little scientific evidence. Our data emphasize the need to further investigate this practice and prospectively investigate the true risks and benefits of reducing loop diuretic therapy in HF patients who seem to be clinically stable based on signs and symptoms only.

A recent article by Felker *et al*.^18^ acknowledges the lack of evidence regarding the correct use of diuretic therapy, despite being an important cornerstone of treatment in HF, irrespective of LVEF. Regardless of the lack of robust evidence, the consensus remains that lower dosages or discontinuation of diuretic therapy should be pursued as stated by Magdy *et al*.^19^ for example. This might be supported by a small randomized controlled trial by Rohde *et al*.^20^ They included 188 stable outpatients with little or no symptoms and a maximum stable furosemide dose of 80 mg. Loop diuretic therapy was withdrawn in 95 of them without occurrence of any safety issues and little need for restarting diuretic therapy. However, the patients in our study were more symptomatic and had more severe HF. This may explain the different outcomes of the study by Rohde *et al*.^20^ and our study. Moreover, some of the patients had clinical signs of volume overload, at least during the first months,^15^ but the increased risk was independent of clinical assessment.

Typically, up‐titration is needed to treat symptoms of congestion due to a destabilization of the disease. Our findings highlight that in approximately one‐third of down‐titration or cessation of diuretic therapy, an escalation in diuretic dosage was required within 30 days, indicating disease destabilization subsequent to down‐titration in a significant number of patients. In some cases, a renewed up‐titration of oral diuretics even seemed to be insufficient to re‐stabilize a patient and further clinical intervention was needed, as indicated in the increased need for (re)hospitalizations. Some patients even died. Thus, while well‐intentioned, down‐titration can potentially precipitate a state of decompensation, which is accompanied with a worse outcome.^21^, ^22^

Indeed, the need for up‐titration was also related to worse outcomes in our study. This may be expected as the intervention may be (too) late and not all hospitalizations can be prevented. Interestingly, poor outcomes—that is, death or hospitalization—occurred in only about 5% within 7 days, suggesting that up‐titration of diuretic therapy was effective in preventing hospitalization in the majority of cases. Little evidence can be found on the effects of up‐titration of (oral) diuretics on the prevention of hospitalizations. A systematic review by Wierda *et al*.^23^ investigates the effects of outpatient treatment with intravenous or subcutaneous diuretics and describes that most studies do not provide satisfactory evidence for a reduction of hospitalization rates. Although their findings do not yield the same suggestions as our results, they also emphasize the need for further prospective studies, mainly to identify patients that would benefit most from changes in diuretic treatment.

A long‐term registry investigating the association between loop diuretic dose changes at baseline and outcomes in chronic HF after 12 months concluded that diuretic dose increase was associated with HF death.^24^ Contrary to our findings, they additionally imply that down‐titration of loop diuretic dose was associated with lower HF and cardiovascular mortality, although the results were not statistically significant. The major difference in their approach is that they only take into account one single dose evaluation moment at baseline, without accounting for any subsequent dose changes that might have occurred closer to the event. In contrast, our study incorporates daily dose data and multiple dose evaluations throughout the analysis period. This methodological difference may explain the contrasting findings between the two studies.

While diuretic dose escalation is widely recognized as a response to worsening congestion and disease progression, often correlating with increased mortality risk,^25^ down‐titration is generally considered a marker of clinical improvement.^24^ However, our findings challenge this assumption, suggesting that patients undergoing diuretic reduction may still be at a heightened risk. This highlights the complexity of interpreting dose adjustments in clinical practice. The observed risk could reflect residual congestion despite dose reduction, or it may indicate a shift in management due to concurrent changes in renal function, blood pressure, or HF therapy. Prior studies have primarily focused on the risks of diuretic intensification, leaving the implications of down‐titration less well understood. Our findings suggest a need for careful patient selection and close monitoring following diuretic reductions, particularly in the absence of standardized criteria to guide these adjustments. Future research should further explore this interplay between diuretic down‐titration, HF progression, and comprehensive treatment strategies that incorporate contemporary HF therapies and precision medicine.

Clinical assessment of fluid status is challenging, and signs and symptoms often occur late.^10^, ^11^ Thus, haemodynamic assessment may identify the need of patients for safe and effective changes in diuretic therapy, although this was not the specific aim of these trials.^10^, ^11^ Also, various biomarkers have been proposed to assess congestion,^26^ as well as imaging tools^27^ and the use of artificial intelligence.^28^ However, there is not sufficient evidence currently that diuretic therapy guidance using these tools may improve outcomes. Our data highlight the need for such specific trials and suggest significant room for improvement in the management of HF patients regarding diuretic therapy.

This study has several limitations that need to be mentioned. Firstly and most importantly, this was a post‐hoc analysis and patients were not randomized to down‐titration versus stable diuretic therapy. Therefore, our results are hypothesis‐generating only. Secondly, a major limitation of our work is the lack of other HF medication usage and dosage of patients during the trial. These can strongly affect the stability of HF patients and influence the decision‐making of clinicians with regard to diuretics. Thirdly, the exact volume status was not known at the time of dose change if done outside of study visits, nor is the application of fluid or salt restriction known for patients. This is of particular importance in the case of renal dysfunction, which may be related to different reasons including both dehydration and volume overload. Also, hypotension may be caused by dehydration or worsening of HF. Thus, it cannot be excluded that down‐titration was done despite volume overload in some cases. Nevertheless, the availability of information on why diuretic doses were changed is a major strength of our study.

It is essential to recognize that the data on which these results are founded dates back several years.^12^ Since the initial study, the drug treatment of HF patients has changed significantly. At the time of TIME‐CHF, the HF guidelines of 2001 and 2005 were in place.^29^, ^30^ Since then, the use of angiotensin receptor–neprilysin inhibitors (ARNIs) in HF with reduced LVEF and SGLT2 inhibitors in both HF with reduced and preserved LVEF have been introduced.^1^ Thus, it is important to confirm our findings in a more current HF population. This is particularly true for the use of SGLT2 inhibition, which might have diuretic effects.^31^ Still, the principles of how to use diuretics in HF have not changed since. We emphasize, however, that due to the retrospective nature of our study, and changing landscape of HF therapy, our results are primarily hypothesis‐generating and need to be validated in new studies incorporating not only the latest of HF therapy but also the growing use of the telemonitoring and eHealth tools.

We should use caution in drawing concrete conclusions about patient outcomes as our dataset does not provide evidence on the effects of diuretic therapy on outcomes. While our study revealed higher mortality rates after dose reduction, it is imperative to underscore the necessity for prospective research to explore the relationship between dose reduction and patient outcomes.

## Conclusion

Re‐evaluation of the current belief in the need for dose reduction of loop diuretics is required. Our study highlights that caution is required if diuretic dosages are reduced in the management of HF patients as currently advised. We have observed a notable risk of needing to reinitiate or increase diuretics within a short timeframe following such reductions. In addition, there may be an increased risk involved. Prospective interventional studies are needed to define the exact role of diuretics in HF and understand the long‐term risks and benefits of adjusting diuretic therapy.

## Supporting information


**Supplementary table 1:** Frequency of events in 30‐day intervals after dose changes, hospital discharge or dose maintenance (EF > 45).
**Supplementary table 2:** Frequency of events in 30‐day intervals after dose changes, hospital discharge or dose; maintenance (EF <= 45) Calibration plot.
