# Supplementary material for: Exploring safety of down‐titrating diuretics in heart failure management
Source: Eur J Heart Fail. 2025 Jul 2;27(8):1393–9. doi: 10.1002/ejhf.3714 (PMC12482844; doi:10.1002/ejhf.3714)
Supplement: Supplementary file 1 — Supplementary table 1: Frequency of events in 30‐day intervals after dose changes, hospital discharge or dose maintenance (EF > 45). Supplementary table 2: Frequency of events in 30‐day intervals after dose changes, hospital discharge or dose; maintenance (EF <= 45) Calibration plot. [file EJHF-27-1393-s001.zip › ejhf3714-sup-0001-table-2.docx]

Supplementary table 2: Frequency of events in 30-day intervals after dose changes, hospital discharge or dose maintenance (EF <= 45)

| **After…** | **N** | **Deceased** | **Hospital admission** | **Down-titration** | **Up-titration** | **No event** |
| --- | --- | --- | --- | --- | --- | --- |
| **Dose maintenance** | 4783 | 0.5% | 1.0% | 7.1% | 7.8% | 83.5% |
| **Downtitration** | 723 | 2.1% | 3.7% | 18.1% | 28.1% | 48.0% |
| **Uptitration** | 748 | 0.8% | 3.5% | 32.9% | 20.3% | 42.5% |
| **Hospital discharge** | 89 | 5.6% | 9.0% | 11.2% | 20.2% | 53.9% |
