# Supplementary material for: Exploring safety of down‐titrating diuretics in heart failure management
Source: Eur J Heart Fail. 2025 Jul 2;27(8):1393–9. doi: 10.1002/ejhf.3714 (PMC12482844; doi:10.1002/ejhf.3714)
Supplement: Supplementary file 1 — Supplementary table 1: Frequency of events in 30‐day intervals after dose changes, hospital discharge or dose maintenance (EF > 45). Supplementary table 2: Frequency of events in 30‐day intervals after dose changes, hospital discharge or dose; maintenance (EF <= 45) Calibration plot. [file EJHF-27-1393-s001.zip › ejhf3714-sup-0001-table-1.docx]

Supplementary table 1: Frequency of events in 30-day intervals after dose changes, hospital discharge or dose maintenance (EF > 45)

| **After…** | **N** | **Deceased** | **Hospital admission** | **Down-titration** | **Up-titration** | **No event** |
| --- | --- | --- | --- | --- | --- | --- |
| **Dose maintenance** | 1286 | 0.8% | 1.3% | 9.5% | 10.3% | 78.1% |
| **Downtitration** | 282 | 1.8% | 2.5% | 14.5% | 34.4% | 46.8% |
| **Uptitration** | 311 | 1.0% | 2.9% | 35.7% | 24.1% | 36.3% |
| **Hospital discharge** | 32 | 0.0% | 6.3% | 28.1% | 21.9% | 43.8% |
